# A comparison of the electrical characteristics, liquid composition, and toxicant emissions of JUUL USA and JUUL UK e-cigarettes

**DOI:** 10.1038/s41598-020-64414-5

**Published:** 2020-04-30

**Authors:** Soha Talih, Rola Salman, Rachel El-Hage, Ebrahim Karam, Sally Salam, Nareg Karaoghlanian, Ahmad El-Hellani, Najat Saliba, Alan Shihadeh

**Affiliations:** 1 Mechanical Engineering Department, Faculty of Engineering and Architecture, American University of Beirut, Bliss Street, P.O. Box 11-0236, Beirut, Lebanon; 2 Chemistry Department, Faculty of Arts and Sciences, American University of Beirut, Bliss Street, P.O. Box 11-0236, Beirut, Lebanon; 3 Center for the Study of Tobacco Products, Virginia Commonwealth University, 821 West Franklin Street, Richmond, Virginia 23284 United States

**Keywords:** Health occupations, Engineering

## Abstract

In 2018, JUUL entered the UK market, where EU regulations limit liquid nicotine concentration to 20 mg/mL, approximately one-third the level of JUUL products sold in the USA. We hypothesized that JUUL’s UK product was engineered to deliver greater electrical power and boost liquid vaporization such that the net nicotine delivery rate was similar to the US version. We compared electrical characteristics, liquid composition, and aerosol emissions of JUUL devices procured in the USA and the UK. Study outcomes included electrical power, total and freebase nicotine, propylene glycol/vegetable glycerin ratio, carbonyls, and reactive oxygen species. Liquids and aerosols were analyzed by GCMS, HPLC, and fluorescence. Compared to the US version, JUUL UK had approximately one-third the liquid nicotine concentration in the liquid (5.4 vs. 1.6 wt.%) and aerosol (4.7 and 1.3 wt.%). Other than nicotine concentration and yield, we found no differences in any other study outcome, including electrical power. Currently, JUUL UK emits nicotine at a far lower rate than the US product, offering an opportunity to study how this factor impacts user behavior, JUUL uptake, and other population-level outcomes across the two markets.

## Introduction

Regulation of electronic cigarettes for public health ends is a growing challenge, particularly as use by previous non-smokers, including children, has risen drastically^1–3^. A central issue for regulation is nicotine delivery to the user. In 2014 the European Union promulgated Directive 2014/40, which limited electronic cigarette liquid nicotine concentration to a maximum of 20 mg/ml^4^. The directive states that this concentration would “allow for a delivery of nicotine that is comparable to the permitted dose of nicotine derived from a standard cigarette.” However, as we have shown previously^5^, nicotine emissions from an electronic cigarette are a function of numerous interacting variables. For example, with all else held constant, nicotine concentration and electrical power can be traded off to attain a given nicotine emission rate per unit time (or “flux”; see Shihadeh and Eissenberg^6^). Because the flux is approximately proportional to the product of electrical power and liquid concentration^5^, a user of a low-nicotine concentration liquid can increase the power of a variable-power electronic cigarette to attain the same or greater nicotine levels as a user of a high nicotine concentration liquid with a lower power device.

JUUL Labs, the producer of the JUUL electronic cigarette, is under investigation by the US FDA for its product design and marketing campaigns^7^, in connection to the widespread uptake of JUUL products by US school children^8,9^. JUUL pioneered the mass marketing of electronic cigarettes loaded with an atypically high nicotine concentration of 60–70 mg/ml^10^. Importantly, the high nicotine concentration aerosol emitted by JUUL was likely made palatable by the fact that the nicotine was emitted in the salt form^11,12^, unlike most other liquids on the market, whose nicotine was predominantly in the harsher free-base form^12–14^. Employing a high nicotine concentration aerosol allowed for the JUUL device to incorporate a relatively low power system with a small battery, enabling the device to exhibit a form factor similar to that of a USB flash drive. It also meant that a given nicotine dose could be delivered to the user in a smaller puff volume, meaning that users could use the device without producing visible aerosol plumes upon exhalation. This feature enabled the practice of “stealth vaping” in classrooms (e.g., drawing a puff when the teacher turns to write on the board) and other places where electronic cigarette use was prohibited^15^.

The introduction of JUUL to the UK in July 2018, where the EU limit on nicotine concentration is in effect, presents an opportunity to observe how a regulation limiting liquid nicotine concentration plays out in the natural environment and may lead to unintended consequences. In this study, we examined how a manufacturer might modify device design to comply with this regulation. We hypothesized that JUUL Labs would increase the power output of its UK devices to compensate for the lower nicotine concentration allowed in that market. A lower nicotine, higher power version of JUUL would in turn translate to greater liquid consumption and toxicant output, resulting in a more hazardous product than the USA device. In this study, we analyzed and compared USA and UK versions of JUUL in terms of electrical power characteristics, liquid and aerosolized nicotine content, and emissions of carbonyl compounds (CCs) and reactive oxygen species (ROS). We found that the power of the UK and USA devices were the same; JUUL had *not* increased the power of the UK version of the device to compensate for its lower nicotine concentration, and apart from lower nicotine yield of the UK version, there were minor differences in measured emissions across the two devices. Thus the null hypotheses held.

## Results

Results are summarized in Table 1. We found that both devices utilized pulse-width modulation to deliver an average of approximately 1.1 V to the pod during puffing. As we previously reported when studying the automatic temperature control feature of JUUL^11^, the power circuit initially delivers 2.7 V for approximately the first half-second of the puff, decaying to a steady value of approximately 1.1 V as the puff proceeds (see Supplementary Fig. S1). Also, both devices automatically cut the power to the pod after a 5.9 sec puff duration has been reached. There were no significant differences in measured resistance of the UK and USA pods. Thus the UK version appears to possess the same key electrical power characteristics as the USA device; in all likelihood, they are the same design.

**Table 1:** Comparison of toxicant emissions, liquid composition and electrical characteristics between JUUL devices sold in the USA and UK. Mean (SD), *p < 0.05, N = 3.

	JUUL USA	JUUL UK	
**Electrical characteristics**			
Max puff duration (sec)	5.86(0.03)	5.92(0.03)	
Average voltage (V)^a^	1.06(0.03)–2.73(0.03)	1.13(0.05)–2.72(0.05)	
Pod resistance (Ω)^b^	1.83(0.06)	1.7(0.1)	
Computed average power (W)	0.62–4.08	0.75–4.36	
**Liquid composition**			
Nicotine concentration (mg/mL)	65(3)	19(1)*	
% Freebase	4.2(2.5)	5.6(2.4)	
pH	6(0.5)	6.5(0.4)	
PG/VG (v/v)	27/73	24/76	
**Emissions in 15puffs**			
Total particulate matter (mg)	27.4(0.46)		33.5(6.51)
Nicotine (mg)	1.3(0.1)	0.4(0.1)*	
Reactive oxygen species (nmol)	1.01(0.36)	0.13(0.005)	
**Carbonyl compounds (µg)**			
Formaldehyde	4.07(0.24)	3.66(0.14)	
Acetaldehyde	3.88(0.37)	3.84(0.47)	
Acetone	13.27(0.1)	13.46(0.29)	
Acrolein	ND	0.01(0.001)	
Propionaldehyde	0.03(0.005)	0.01(0.03)	
Crotonaldehyde	0.4(0.02)	0.4(0.02)	
Methacrolein	1.54(0.02)	1.54(0.01)	
Butyraldehyde	ND	ND	
Valeraldehyde	ND	ND	
Glyoxal	0.35(0.004)	0.36(0.007)	
Methylglyoxal	2.08(0.21)	1.16(0.33)*	
Total carbonyls	25.71(0.55)	24.48(0.3)*	

^a^Voltage delivered to the coil was found to vary during a 6 sec puff, with a maximum average of 2.73(0.04)V for both devices during the first 0.6 sec. The voltage stabilizes after that to an average of 1.09(0.05)V. ^b^Resistance refers to that of the coil at room temperature; the resistance of the coil is expected to change as its temperature increases during puffing.

JUUL UK pods had a nicotine concentration of approximately one-third that of JUUL USA (19 mg/mL vs. 65 mg/mL, p < 0.001). For both versions, the nicotine was nearly entirely in the protonated form, and the liquid solvent was approximately 25/75 PG/VG (v/v); there were no significant differences in these measures across the US and UK devices. Thus, the only difference noted in device design across UK and USA JUUL was the nicotine concentration of the liquid.

In 15 puffs, mean total particulate matter emitted by each device was the same (approximately 30 mg), but nicotine yield of JUUL USA was approximately three times that of JUUL UK (1.3 mg vs. 0.4 mg, p < 0.001), mirroring the difference in liquid nicotine concentration. Apart from methylglyoxal, both JUUL versions were found to emit similar levels of CCs, at much lower levels than found in combustible cigarettes (total CCs 25 µg/15 puffs vs. 2000 µg/combustible cigarette^11^). JUUL USA exhibited approximately double the methylglyoxal as JUUL UK, accounting for the small but significant difference in total CCs observed across the two devices. ROS emissions were not significantly different across devices, and were much lower than ROS emitted by a single combustible cigarette (less than 1 nmol/15 puffs vs. 25nmol/combustible cigarette^16^). Thus by virtue of the lower nicotine yield, JUUL UK’s toxicant emissions are approximately three times those of JUUL USA, per unit nicotine emitted.

## Discussion

JUUL devices sold in the UK were generally found to be the same in every measure as those sold in the USA except for nicotine liquid concentration and yield, which were approximately one-third those of the USA version. At the time these products were procured, JUUL Labs thus appeared to have complied with EU restriction on nicotine content, without re-engineering the device or liquid itself, apart from reducing nicotine concentration. The hypothesis that JUUL UK would have greater electrical power failed.

These findings thus highlight an ongoing natural experiment on the manipulation of nicotine content in what is likely the world’s most popular electronic cigarette product. Observation of JUUL user behavior in the UK and USA may provide valuable insights on how population characteristics, toxicant exposure, pod consumption, and reported health effects vary when nicotine concentration is regulated.

For example, users of JUUL UK devices may adjust their puffing patterns to obtain similar levels of nicotine as obtained with the JUUL USA devices. It has been reported that when given low nicotine concentration liquids, electronic cigarette users increased puff frequency, duration and liquid consumption^17^, and the more intensive puffing regimen associated with the reduced nicotine liquids resulted in higher measured carbonyl emissions^18^. This factor and the results from the current study would, therefore, suggest that to the extent that users seek a given nicotine dose, exclusive users of JUUL devices may be exposed to three times the CC and ROS emissions when using JUUL UK relative to JUUL USA.

## Methods

We analyzed and compared liquid composition (nicotine concentration, pH, and PG/VG ratio), and emissions (nicotine, CCs, and ROS) of tobacco flavored JUUL pods procured in the USA and the UK using previously described methods^5,13,16,19^. In brief, for each device aerosol was generated using the AUB Aerosol Lab Vaping Instrument (ALVIN), programmed to execute 15 puffs of 4 sec duration, 10 sec interpuff interval and 1 L/min flow rate^20^. The aerosol exiting the ECIG was trapped on glass fiber filters, and nicotine in the aerosol was extracted and analyzed for protonated and free-base fractions using a liquid-liquid extraction method and GCMS, as in El-Hellani, *et al*.^13^. We note that Duell *et al*.^12^ suggest that the method described in El-Hellani *et al*. may be biased due to dilution and re-equilibration phenomena during the extraction process, however because we work with nicotine concentrations greater than 600 μg/mL, the maximum bias introduced by our analytical method is 0.15% for the worst-case scenario of a highly acidic ECIG liquid, as we have shown^13^.

CCs were trapped on DNPH cartridges, eluted with 90/10 (vol/vol) ethanol/acetonitrile and quantified by HPLC-UV, as in El-Hellani, *et al*.^19^. ROS reported as H_2_O_2_ equivalent were analyzed by immersing the particulate filter pads in 20 mL of freshly prepared DCFH probe solution. Fluorescence was read on a SpectraMax M5 microplate reader acting as a fluorimeter, as in Haddad, *et al*.^16^. All analyses were performed in triplicate, using a new pod for each puffing bout.

JUUL electrical power characteristics were determined by disassembling the power unit of each device and connecting the JUUL power control circuit output leads to a data acquisition device sampling at 20 kHz while drawing three consecutive 10 sec duration puffs at 1 L/min via ALVIN. Also, the resistance of 3 different pods from each the UK and USA devices were measured at ambient temperature (25 °C) using a laboratory Ohmmeter.

## Supplementary information

Figure S1.
