# Supplementary material for: A comparison of the electrical characteristics, liquid composition, and toxicant emissions of JUUL USA and JUUL UK e-cigarettes
Source: Sci Rep. 2020 Apr 30;10:7322. doi: 10.1038/s41598-020-64414-5 (PMC7192936; doi:10.1038/s41598-020-64414-5)
Supplement: Supplementary file 1 — Figure S1. [file 41598_2020_64414_MOESM1_ESM.docx]

**Supplementary Information**

**A comparison of electrical characteristics, liquid composition, and toxicant emissions of
JUUL USA and JUUL UK e-cigarettes**

Soha Talih, PhD; Rola Salman, BS; Rachel El-Hage, MS; Ebrahim Karam, BE; Sally Salam, BS; Nareg Karaoghlanian, BE; Ahmad El-Hellani, PhD; Najat Saliba, PhD**;** Alan Shihadeh, ScD

**Supplementary Figure S1**


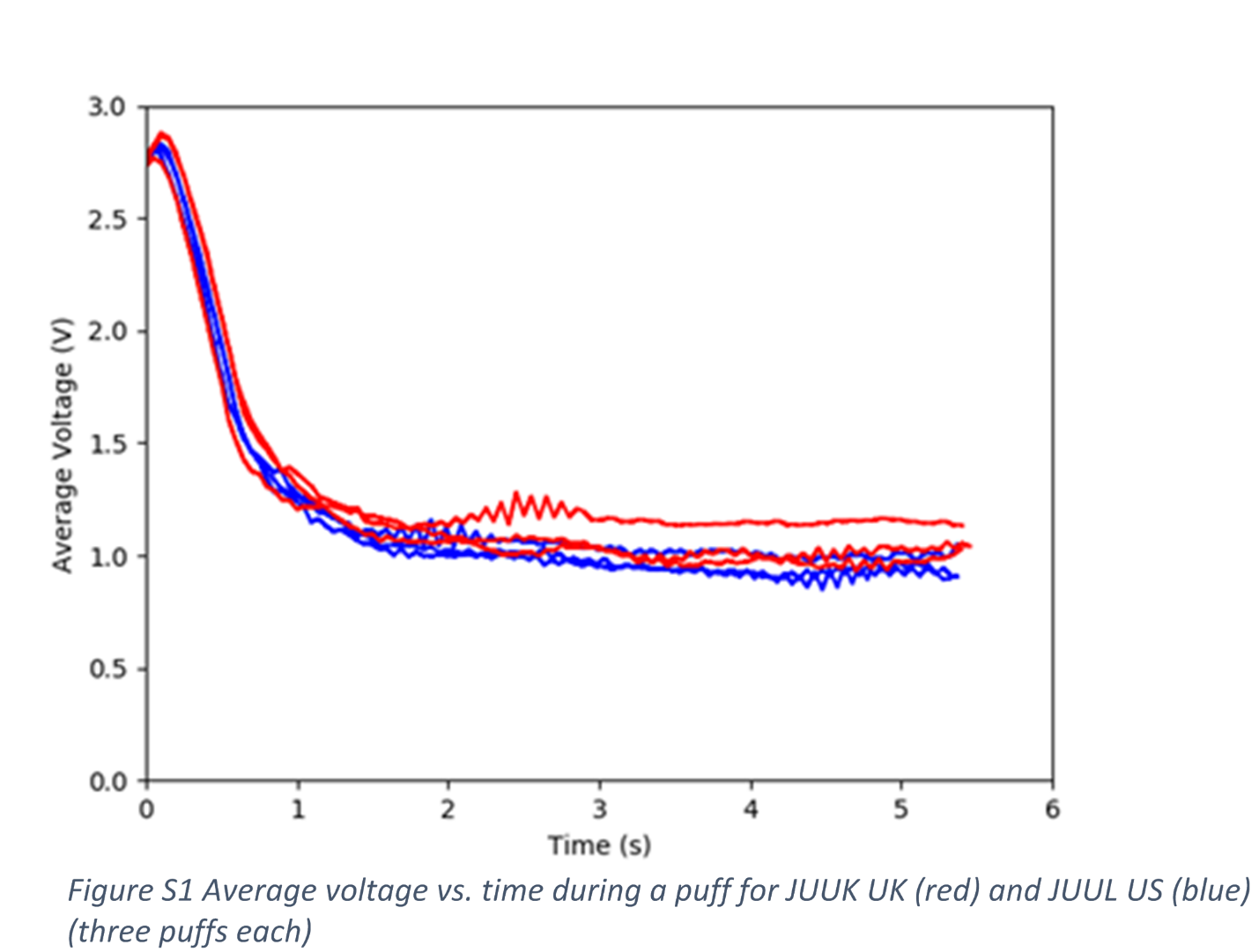


Figure S1 Average voltage vs. time during a puff for JUUL UK (red) and JUUL US (blue) (three puffs each)
